# Recent Developments in Cell Permeable Deubiquitinating Enzyme Activity-Based Probes

**DOI:** 10.3389/fchem.2019.00876

**Published:** 2019-12-18

**Authors:** Daniel Conole, Milon Mondal, Jaimeen D. Majmudar, Edward W. Tate

**Affiliations:** ^1^Department of Chemistry, Imperial College London, London, United Kingdom; ^2^Medicine Design, Pfizer Inc., Cambridge, MA, United States

**Keywords:** deubiquitinase, cell permeability, activity based probe, small molecule, DUB activity, ubiquitin (Ub), deubiquinating enzymes

## Abstract

Deubiquitinating enzymes (DUBs) function to remove or cleave ubiquitin from post-translationally modified protein substrates. There are about 100 known DUBs in the proteome, and their dysregulation has been implicated a number of disease states, but the specific function of many subclass members remains poorly understood. Activity-based probes (ABPs) react covalently with an active site residue to report on specific enzyme activity, and thus represent a powerful method to evaluate cellular and physiological enzyme function and dynamics. Ubiquitin-based ABPs, such as HA-Ub-VME, an epitope-tagged ubiquitin carrying a C-terminal reactive warhead, are the leading tool for “DUBome” activity profiling. However, these probes are generally cell membrane impermeable, limiting their use to isolated enzymes or lysates. Development of cell-permeable ABPs would allow engagement of DUB enzymes directly within the context of an intact live cell or organism, refining our understanding of physiological and pathological function, and greatly enhancing opportunities for translational research, including target engagement, imaging and biomarker discovery. This mini-review discusses recent developments in small molecule activity-based probes that target DUBs in live cells, and the unique applications of cell-permeable DUB activity-based probes vs. their traditional ubiquitin-based counterparts.

## Introduction

### The Ubiquitin-Proteasome System

The ubiquitin-proteasome system (UPS) has attracted more excitement, scope and promise as a therapeutic target than any system since the rise of the kinome as a druggable protein family. This biological process regulates proteolysis, transcriptional regulation, DNA damage, complex formation, cellular trafficking and localization, inflammation and autophagy, therefore modulation of ubiquitin-proteasome pathways are a potentially rich source of new therapeutic modalities (Fleury and Walker, 2015; Hewings et al., 2017). The key post-translational modification (PTM) in this pathway is ubiquitination, which is catalyzed by the E1–E2–E3-enzyme cascade resulting in isopeptide coupling of a ubiquitin (Ub) C-terminus primarily to a lysine residue of an acceptor protein (Glickman and Ciechanover, 2002). The ubiquitin is then itself elongated to form various branched or linear polyubiquitin chains which, depending on their topology, may lead to varied functional outcomes (Elias et al., 2003; Swatek and Komander, 2016; Haakonsen and Rape, 2019).

### Deubiquitinase Enzymes: Function and Importance

In line with the importance of ubiquitination for regulation of many cellular processes, the human genome encodes about 100 deubiquitinating enzymes (DUBs) that can reverse this PTM by hydrolysing the amide bond between mono- and poly-Ub chains, and substrate proteins (Hewings et al., 2017; Clague et al., 2019). Similarly to Ub ligases, DUBs thus regulate protein activity, stability, localization, and interactions (Fleury and Walker, 2015). Although less extensively studied than the much larger class of Ub ligases (numbering over 600), DUBs have attracted intense attention in recent years as promising targets for drug development in various indications, particularly in cancer (D'Arcy and Linder, 2014; D'Arcy et al., 2015). However, significant challenges remain in the identification of selective ligands for DUBs, which would in turn aid in the determination of dynamic DUB substrate profiles among the tens of thousands of Ub sites and diverse Ub polymer topologies, distributed across the majority of proteins in the cell.

### DUB Activity-Based Probes

#### A Brief History

**Figure 1:**
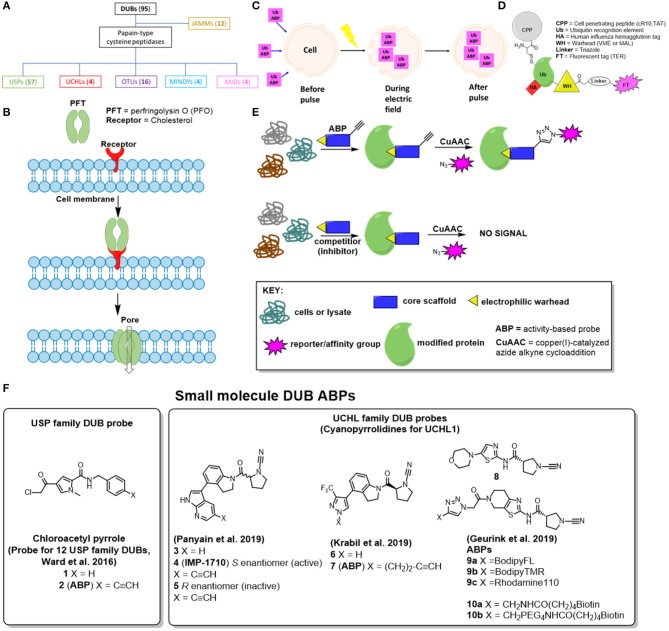
Visual representations of different approaches to address the cell permeability challenge associated with developed DUB ABPs. **(A)** Family tree for DUBs, parenthesis displays how many enzymes are in this sub-family, **(B)** Pore-forming toxins, **(C)** Electroporation, **(D)** Cell penetrating peptide-based DUB ABPs **(E)** Activity-based protein profiling with small molecule-based DUB ABPs—**(F)** Small molecule DUB inhibitors from the literature that have been converted to ABPs.

To better understand the function and mechanism of these DUBs, activity-based probes (ABPs) have been developed over the last two decades. There are five DUB sub-types consisting of USPs (ubiquitin-specific proteases), UCHs (ubiquitin carboxy-terminal hydrolases), MJDs (Machado–Josephin domain-containing proteases), OTUs (ovarian tumor proteases) and MINDYs (motif- interacting with ubiquitin-containing novel DUB family) that are papain-type cysteine peptidases (Figure 1A). These DUBs possess a catalytic nucleophilic cysteine residue that can be captured covalently by reaction with an electrophilic warhead based ABP (Harrigan et al., 2017; Hewings et al., 2017). Appending a reporter tag to the electrophilic warhead creates an ABP which can inform on DUB selectivity and proteolytic activity, and facilitate novel inhibitor profiling. Distinct from these families are JAMMs (JAB1, MPN, MOV34 family), which are zinc metallopeptidases which are not as well-understood but are likely to require different chemistries for ABP development.

The first generation and most widely employed DUB ABPs contain a mono-Ub recognition element with either a propargylamide or vinyl methyl ester electrophilic group conjugated to the C-terminus of Ub (Borodovsky et al., 2002; de Jong et al., 2012; Ekkebus et al., 2013), and a fluorescent reporter group for detection of the labeled enzyme (Fleury and Walker, 2015; Leestemaker and Ovaa, 2017). Later, this type of ABP was extended to include internal and terminal di-ubiquitin as the targeting element to provide insight into the linkage specificity of DUBs and the nature of their binding interaction with protein substrates (McGouran et al., 2013; Li et al., 2014; Mulder et al., 2014; Flierman et al., 2016).

More recently, a more sophisticated Ub-based ABP incorporated methyl disulphide as the reactive warhead, which allows the release of active DUBs from the ABP under mild conditions, such that they can be isolated from complex cell extracts for further study (de Jong et al., 2017; Leestemaker and Ovaa, 2017).

#### In-cell Profiling: A New Frontier for DUB Biology

While these various generations of DUB ABPs are widely used and have greatly advanced our knowledge regarding the biological role of DUBs, they can only be employed on cell lysates as the large size of the Ub recognition element(s) precludes cellular permeability (Hewings et al., 2017). Cell lysis causes cytoplasmic and nuclear protein dilution, and disruption of cellular organization and localization, leading to dissociation of important protein-protein interactions (PPIs) necessary for DUB activity and dysregulation of ubiquitination patterns (Claessen et al., 2013; Gui et al., 2018). The consequence is that traditional Ub-based ABPs have limited use for the exploration of dynamic DUB activity profile (Fleury and Walker, 2015), in common with the well-known differences in protease activity profiles measured between lysates and live cells (Hewings et al., 2017).

In order to gain a full understanding of the function of DUBs in the most relevant cellular and physiological setting, the issue of cellular permeability needs to be addressed. Comprehensive reviews of Ub-based ABPs have recently been published and thus the present review focuses on recent work toward cell permeable DUB ABPs (Fleury and Walker, 2015; Hewings et al., 2017; Leestemaker and Ovaa, 2017).

## Efforts to Address DUB ABP Cell Permeability

Attempts to date to address cell-penetration for DUB ABPs can be divided into four categories: pore-forming toxins (Claessen et al., 2013), electroporation (Mulder et al., 2016), cell-penetrating peptide (CPP) ABPs (Gui et al., 2018) and small-molecule based cell permeable ABPs (Ward et al., 2016, 2019; Geurink et al., 2019; Krabill et al., 2019; Panyain et al., 2019). These are discussed in further detail below and in Figure 1.

### Pore-Forming Toxins

In 2013, Claessen et al. published a catch-and-release Ub ABP to map the endogenous expression of DUBs and their interacting proteins in semi-intact cells (Claessen et al., 2013). The catch component consisted of a biotin affinity handle, whereas the release motif entailed a cleavable linker (either hydrazine, azobenzene, or levulinoyl ester), accessed through a combination of intein chemistry and sortase-mediated ligation. While this probe improved DUB peptide detection by mass spectrometry (MS), it remained, like those before, cell impermeable. To combat this, the authors of the paper employed perfringolysin O (PFO), a soluble toxin secreted by the pathogen *Clostridium perfringens* that binds cholesterol and forms large homo-oligomeric pore complexes to allow the ABP to cross the cell membrane into the cytosol (Figure 1B). Interestingly, they identified 34 DUBs and their interacting partners in non-infected cells, and three additional host DUBs (USP36, USP33, and TRABID) in *chlamydia*-infected HeLa cells that were not detected previously using Ub-VME based ABPs. However, a head-to-head quantitative proteomics analysis (live cells vs. cell lysate) to further support this claim was not undertaken in this study, and this approach has not been widely adopted by other labs.

### Electroporation

In another report, a cascading activity-based probe, Ub-Dha (Ub-dehydroalanine) was used to monitor catalysis along the E1, E2, and E3 enzyme trans-thioesterification reaction pathway (Mulder et al., 2016). This probe aimed to capture a dynamic post translational pathway, and although the probe was not designed to interact with DUBs, the approach to deliver it across the cell membrane is relevant for DUB ABP design. To this end, the authors used electroporation, an electrical pulse applied to cells to temporarily induce cell membrane micropore formation (Figure 1C). While generally used to transfect exogenous DNA into cells, this technology may be useful for the intracellular delivery of large molecules, such as Ub-Dha (Shi et al., 2018). An advantage of this method, particularly in comparison to pore-forming toxins, is that the pores formed are very small and transient, so cell viability and functionality is usually preserved (Mukherjee et al., 2018). Consistent with this, the authors report normal cell morphology post-electroporation.

In-gel fluorescence studies showed that labeling of key Ub E1 enzymes (UBA6 and UBE1) in live cells using electroporation occurred on a similar timescale to that in lysates. Also, UBE1 activity could be attenuated with pre-treatment of lysates or live cells with PYR-41 (a small molecule UBE1 inhibitor), further suggesting that electroporation was successfully delivering Ub-Dha into cells. While this method was focused primarily on capturing Ub ligases, four DUBs were also labeled and identified using MS-based quantitative proteomics in HeLa cells. This work was a landmark in Ub ligase profiling, but no data were provided to show whether electroporation in live cells produces significantly different results in DUB labeling compared with that in cell lysates, and the method has not been widely taken up.

### Cell-Penetrating Peptide (CPP) Based ABPs

The Zhuang group recently described cell-permeable DUB ABPs consisting of various combinations of polycationic cell-penetrating peptides (CPPs) conjugated to ubiquitin and thiol-reactive warheads (Figure 1D; Gui et al., 2018). Chemoselective ligation was employed to attach either a cyclic polyarginine (cR10) or KRKKRRQRRR (TAT) peptide to the Ub N-terminus, whereas propargylamine (PA) or vinyl methyl ester (VME) were used as the electrophilic warhead (Ekkebus et al., 2013). In addition, a disulphide bond was built-in to allow reductive release of the CPP from the ABP once it had entered the cell, and a human influenza hemagglutinin (HA) tag was incorporated for affinity purification. Tetraethyl-rhodamine (TER) fluorescent versions of these ABPs were also synthesized to demonstrate live-cell uptake of CPP-containing probes by live-cell fluorescence confocal microscopy. Importantly, the authors synthesize and evaluate appropriate control probes (without the CPP motifs or thiol-reactive warheads) to demonstrate that the difference in observed live cell DUB labeling can be attributed to these DUB ABP elements.

The authors then profiled the DUBome using HA-tagged ABPs in live cells using immunoblotting and quantitative mass spectrometry proteomic analysis. With respect to immunoblotting, the band profile for live cell DUB labeling with the HA-Cys(cR10)-Ub-PA probe was notably different to that of cell lysates. Using a label-free quantitative proteomics method, 34 DUB proteins were identified after treatment of live HeLa cells with the HA-Cys(cR10)-Ub-PA probe, and 27 of these were found to be significantly enriched [log_2_ (fold difference) >2, *p*-value < 0.05]. Importantly, the authors provide a proteomic level comparison of this probe in live cells vs. cell lysates and demonstrate that treatment of the cell lysate with HA-Cys(cR10)-Ub-PA probe followed by equivalent sample processing results in identification of only 10 DUBs, of which all were also detected in the live cell experiment. Interestingly, the live cell labeling experiment identified DUBs that are present in different organelles, suggesting that the probe is permeating various sub-cellular compartments. Finally, pan-DUB inhibitor PR-619 was employed to test whether these probes could be used for live cell DUB profiling studies and novel DUB inhibitor discovery. PR-619 inhibited intracellular DUB activity in a concentration-dependent manner, and labeling was more pronounced in lysates vs. live cells. This result suggests the nuances that may be missed if DUB activity is not measured in a physiological relevant system.

### Small Molecule ABPs

In 2016, Ward et al. published the first small-molecule based DUB ABP, based on a chloroacetylpyrrole scaffold (**1**), which was originally identified from a high-throughput screening (HTS) campaign at Mission Therapeutics (Figure 1F; Ward et al., 2016). This compound exhibited potent USP4 and USP11 biochemical activity, and so the authors employed a competitive activity-based protein profiling (ABPP) method to assess whether an alkyne-tagged analog **2** could be used as a live cell DUB ABP for quantitative target engagement. Intriguingly, **2** labeled a total of 12 DUBs in U2OS cells. Furthermore, it was discovered that parent chloroacetylpyrrole (**1**) could compete against alkyne-tagged probe (**2**) in a concentration dependent manner for at least 9 different DUBs, in some cases at sub micromolar concentrations, which is noteworthy considering its structural simplicity. While a direct quantitative mass spectrometry comparison with cell lysate labeling is not reported, the authors demonstrate using a Ub-Rhodamine fluorescent intensity assay that parent chloroacetylpyrrole (**1**) biochemically inhibits 11 of these DUBs at EC_50_ values <10 μM.

As one might expect with a small molecule based ABP, probe **2** also targets many non-DUB proteins, presumably through numerous non-specific reactions with reactive cysteine residues (Ward et al., 2016; Hewings et al., 2017). While this could complicate studies that aim to link a specific function or phenotype to DUB target engagement, the small molecule ABPs still serves as a useful—and to date unique—tool for assessing cellular target engagement and selectivity of novel DUB inhibitors.

More recently, an ABPP approach was also employed to ascertain the molecular explanation for the cellular toxicity of VLX1570 (analog of b-AP15), a small molecule USP14 inhibitor for refractory multiple myeloma that has been put on full clinical hold due to dose limiting toxicity (Ward et al., 2019). The authors prepare an alkyne-tagged version of VLX1570 (structure not shown), and through various immunoblotting and proteomics experiments show high protein target promiscuity, the formation of higher molecular weight complexes, and resultant aggregation and inhibition of CIAPIN1, an important anti-apoptotic protein. This work highlights the importance of determining the protein target activity profile for drug candidates as part of the drug discovery process.

Several potent and selective small molecule covalent inhibitors have emerged for DUBs in the USP and UCHL subfamilies, including in the patent literature (Kemp and Woodrow, 2018; Kemp et al., 2018; Gibson et al., 2019a,b) and these scaffolds present a potential opportunity to design novel cell permeable ABPs. Taking this approach, Panyain et al. in collaboration with Mission Therapeutics designed IMP-1710 (**4**), a highly potent and selective cyanopyrrolidine ABP against UCHL1, with 40 nM IC_50_ and sensitive detection down to 2 nM ABP in a range of cell types (Figure 1F; Panyain et al., 2019). Extensive biochemical, quantitative proteomic and Ub-based probe profiling demonstrated exquisite activity-dependent selectivity for UCHL1 over all other DUBs, and a highly favorable selectivity profile at the whole proteome level. Interestingly, IMP-1710 (**4**) is highly stereoselective, with opposite enantiomer **5** providing an effective inactive control, and could be used to show that the small molecule LDN-57444, previously reported as a UCHL1 tool inhibitor and widely used in the literature, fails to engage UCHL1 biochemically or in cells (Liu et al., 2003). Finally, the authors used compound **3** and IMP-1710 (**4**) to demonstrate the therapeutic potential of UCHL1 inhibition in a model of idiopathic pulmonary fibrosis, without cytotoxicity. Related alkyne-tagged small molecule ABP **7** of cyanopyrrolidine **6** was recently reported by Krabill et al., although this molecule is >150-fold less potent than IMP-1710 (**4**), and is relatively non-specific (Figure 1F; Krabill et al., 2019).

In addition, Geurink et al. recently reported some fluorescently labeled (**9a-9c**) and biotinylated (**10a**, **10b**) ABPs of related cyanopyrrolidine-based scaffold **8** (Figure 1F; Geurink et al., 2019). Through ABPP mass spectrometry the authors demonstrated strong UCHL1 selectivity within the DUB family, with PARK7 - also known as DJ-1, a neuroprotective redox-sensitive chaperone—observed as a majority off-target protein. Additional off-targets were observed by gel electrophoresis at ~55 kDa; these were not identified by the authors, but based on the work of Panyain et al. these off-targets are likely to be aldehyde dehydrogenases (ALDH). Although the authors demonstrate imaging of probe-labeled proteins in zebrafish embryos, strong off-target labeling of DJ-1 and ALDH may limit the utility of these probes in living systems.

## Discussion

### Learning From Other Target Classes

Ubiquitin based ABPs have significantly advanced our knowledge of DUB structure, dynamics and function. However, their general inability to cross the cell membrane prevents further understanding concerning DUBs in their native, physiological environment (Fleury and Walker, 2015; Ward et al., 2016; Hewings et al., 2017). The potential applications of cell-permeable DUB ABPs reaches beyond improved understanding of DUBs within a given cell or model organism. Development of cell permeable DUB ABPs may lead to agents that allow direct visualization and quantification of DUB activity in living organisms, and may even extend to the development of DUB-based assays for DUB activity as a clinical biomarker, as well as a tools for preclinical *in vivo* and clinical *ex vivo* evaluation of DUB inhibitors and target engagement (Fleury and Walker, 2015). Basic and translational studies on other hydrolase classes including serine proteases, lipases, caspases, and cathepsins have benefitted greatly from the development of cell permeable ABPs, where significant and sustained research has led to ground-breaking non-invasive *in vivo* imaging probes (Blum et al., 2007; Edgington et al., 2009). In the proteasome field, development of cell permeable ABPs for proteasome and immunoproteasome catalytic subunits such as Dansyl-Ahx3-L3-VS (Berkers et al., 2005) and BodipyFL-Ahx3-L3-VS (Berkers et al., 2007) have become popular tools for in-gel fluorescence imaging, flow cytometry, and fluorescence microscopy in animal tissues (Gan et al., 2019).

The development of small molecule DUB probes is thus a high priority for the field in order for DUB ABPs to match the utility of their counterparts in these other target classes, particularly for *in vivo* applications.

### Comparing the Approaches

For a probe to be used as a true ABP, it must be able to capture the protein in its native environment in a strictly activity-dependent manner, features for which cell permeability is critical, ideally through passive diffusion or native uptake mechanisms. Furthermore, an ideal ABP for determining both cellular target engagement and selectivity of novel DUB inhibitors should label a large spectrum of DUBs at a low concentration, preferably 1 μM or lower. Most of the methods reviewed here except a few of the small molecule approaches employ large amounts of probe (>10 μM), precluding their use for cellular discovery of novel reversible or irreversible DUB inhibitors with a weak K_I_ component (Claessen et al., 2013; Mulder et al., 2016; Gui et al., 2018). Small molecule ABPs, particularly those which cover a majority of the DUBome, would check most of these boxes since they could be used in low concentrations on live cells for short periods of time, enhancing identification of novel DUB inhibitors and our understanding DUB biological functions under various (patho)physiological contexts (Table 1).

**Table 1:** Comparison of various approaches to DUB ABP cellular permeability with respect to the requirements for an ideal DUB ABP.

**ABP**	**Methods of cell entry**	**Requirements for an ideal DUB ABP**		
		**Proteins captured in their native environment?** **(Yes/No/Partial)**	**ABP labels a large spectrum (>10) of DUBs** **(Yes/No/Partial)**	**Can use low (<1 μM) con-centrations of ABP?** **(Yes/No/Partial)**
Traditional Ubiquitin-based e.g., HA-Ub-VME	None	No	Yes	No
	Pore-forming toxins	Partial	Yes	No
	Electroporation	Partial	Partial	No
	Cell-penetrating peptides	Partial	Yes	No
Small molecules	Passive diffusion	Yes	Partial	Yes

Ub-based ABPs possess a distinct advantage over peptide and small molecule based ABPs because of the specificity of their recognition element, and shortening the recognition element to allow greater cell permeability fails to preserve specificity or activity (Albrow et al., 2011; Safa et al., 2019). Each of the various approaches discussed above to force entry of Ub-based ABPs, including toxin pore formation, electroporation and so-called “cell-penetrating” peptides (CPPs), raise numerous concerns regarding host membrane repair responses that may be triggered as an unintended consequence (Ostolaza et al., 2019). For example, CPPs have been demonstrated to substantially disrupt membrane integrity, causing formation of non-physiological subcellular compartments (Gao et al., 2019). Consequently, there is a chance that upon pore formation that cell homeostasis is disrupted, and either apoptosis, necroptosis or pyroptosis results, which would most likely only be compounded in an *in vivo* environment due to cell signaling (Abdelrazzak et al., 2011; Etxaniz et al., 2018). Each of these approaches also present significant challenges for extended periods of live cell profiling, and are largely inapplicable to whole organism analysis (Shi et al., 2018). CellSqueeze technology, which uses a commercial microfluidics device and pressure system to open membrane pores, may provide an alternative approach to garner higher value from cell-impermeable probes (Szeto et al., 2015; Li et al., 2017). Whether this would improve DUB coverage or better preserve cell integrity remains to be seen, and the method may be difficult to scale.

Small molecule based ABPs, particularly those designed with an alkyne or other biorthogonal enrichment handle for ABPP, can passively diffuse through the cell membrane to afford selective labeling, visualization, and enrichment of active enzymes in a complex proteome without disruption to cellular organization (Martell and Weerapana, 2014; Fleury and Walker, 2015). To date there have been remarkably few successful reports of small molecule DUB ABPs, but two recent examples of a pan-USP ABP and a highly UCHL1-specific ABP have shown the promise of this approach, generating significant interest in the DUB field (Ward et al., 2016; Akinjiyan et al., 2017; Hewings et al., 2017; Wong et al., 2017; Panyain et al., 2019). Issues including probe specificity, DUB spectrum, toxicity and metabolic stability will need to be addressed in order to realize the full potential of cell permeable DUB ABPs and their applications to sophisticated *in vivo* studies such as imaging and biomarker analysis. Focused research from the medicinal chemistry and chemical biology communities, as well as close collaboration with industry partners with deep expertise in DUB inhibitor discovery, will continue to play a key role in this endeavor.

## Author Contributions

DC prepared the manuscript. JM, ET, and MM reviewed the manuscript and provided comments, suggestions and edits.

### Conflict of Interest

ET is a founder, shareholder and Director of Myricx Pharma Ltd. JM is an employee of Pfizer Inc. The remaining authors declare that the research was conducted in the absence of any commercial or financial relationships that could be construed as a potential conflict of interest.
